# The causal role of gastroesophageal reflux disease in anxiety disorders and depression: A bidirectional Mendelian randomization study

**DOI:** 10.3389/fpsyt.2023.1135923

**Published:** 2023-02-22

**Authors:** Youjie Zeng, Si Cao, Heng Yang

**Affiliations:** ^1^Department of Anesthesiology, Third Xiangya Hospital, Central South University, Changsha, Hunan, China; ^2^Department of Neurology, Third Xiangya Hospital, Central South University, Changsha, Hunan, China

**Keywords:** gastroesophageal reflux disease, anxiety disorders, depression, causal relationship, incidence risk, GWAS, single-nucleotide polymorphisms, Mendelian randomization

## Abstract

**Background:**

Observational studies have shown an association between gastroesophageal reflux disease (GERD) and anxiety disorders/depression. However, these evidences may be influenced by confounding factors. Therefore, our study aimed to determine the causal relationship between GERD and anxiety disorders/depression by conducting a bidirectional Mendelian randomization (MR) study.

**Methods:**

We performed a bidirectional MR analysis using summary statistics from genome-wide association studies (GWAS) in European individuals. The inverse-variance weighted (IVW) method was used as the primary analytical method to assess causality. In addition, five additional MR methods [maximum likelihood, MR-Egger, weighted median, robust adjusted profile score (MR-RAPS), and mode-based estimate (MR-MBE)] were performed to supplement the IVW results. Furthermore, several sensitivity analyses were performed to assess heterogeneity, horizontal pleiotropy, and stability. Finally, a multivariable MR (MVMR) analysis was performed to determine the causal relationship by adjusting for potential confounders.

**Results:**

MR results of the IVW method indicated that GERD significantly increases the risk of anxiety disorders [odds ratio (OR) = 1.35, 95% confidence interval (CI) 1.15–1.59, *P* = 2.25 × 10^–4^] and depression (OR = 1.32, 95% CI: 1.15–1.52, *P* = 1.26 × 10^–4^). In addition, the MR results of maximum likelihood, MR-Egger, weighted median, MR-RAPS, and MR-MBE remained parallel to the IVW results. Furthermore, sensitivity analysis suggested that the results were robust, with no pleiotropy or heterogeneity detected. Nevertheless, reverse MR analysis showed that anxiety or depression did not increase GERD risk. Finally, MVMR analysis showed that the effect of GERD on increasing the risk of anxiety disorders/depression was independent of confounders.

**Conclusion:**

This MR study supports a causal association between GERD and an increased risk of anxiety disorders and depression. Therefore, complementing symptomatic treatment of GERD with psychological assessment and necessary psychological support therapy may help reduce the risk of future anxiety disorders and depression.

## 1. Introduction

Anxiety disorders and depression are the most prevalent mental disorders on a global scale, which impose a substantial social and economic burden (1, 2). In addition, anxiety disorders and depression are characterized by impairments in behavioral and affective indicators of social functioning (3). Moreover, individuals with anxiety disorders and depression are more likely to suffer from chronic comorbidities, which may exacerbate psychological impairment (4, 5). Overall, as the quality of life of persons with anxiety disorders and depression can be substantially affected, it is vital to promote strategies for preventing anxiety disorders and depression.

Gastroesophageal reflux disease (GERD) refers to symptoms or complications triggered by the reflux of acidic stomach contents into the esophagus (6). It is estimated that roughly 20% of adults in the Western world suffer from GERD (7). A large cross-sectional observational study has revealed that, compared with healthy control individuals, GERD patients experienced significantly higher anxiety and depression levels (8). In addition, a study conducted on Japanese GERD patients treated with proton pump inhibitors (PPI) showed that the PPI partial responder group had significantly higher Anxiety and Depression Scale scores compared to the PPI responder group (9). Furthermore, a population-based cohort study conducted in Taiwan showed that the GERD cohort had a higher risk of anxiety disorders and depression than the control cohort (10). The study also revealed a higher rate of newly diagnosed cases of anxiety disorders and depression throughout all the follow-up periods (10). Although the underlying mechanisms are not fully understood, several pieces of current evidence may support GERD-induced anxiety disorders and depression. The elevated global inflammation levels triggered by GERD may contribute to the development of anxiety and depression (10). In addition, more than half of patients with chronic GERD have nighttime acid reflux, which severely interferes with rest, increasing anxiety and tension (11, 12). In turn, studies also suggest that anxiety or depression may increase GERD risk (13). Subjects with both anxiety and depression had a 2.8-fold increased reflux risk compared to healthy control individuals (14). Kessing et al. reported that the degree of anxiety was correlated with the severity of reflux episodes (15).

Despite evidence suggesting a possible association between GERD and anxiety disorders/depression, these observational studies cannot explain cause and effect. Therefore, it is currently unclear regarding the causal association between GERD and anxiety disorders/depression. The Mendelian randomization (MR) study, similar to randomized controlled trials (RCT), is an innovative research approach for investigating the causal relationship between exposure and outcome (16). In MR research, single-nucleotide polymorphisms (SNPs) highly correlated with exposure were utilized as instrumental variables (IVs) to evaluate the causal association between exposure and outcome (17). SNPs conform to the principle of random assignment of genetic variants at meiosis, which minimizes the influence of confounding variables and the possibility of reverse causation since genetic variants precede disease occurrence (18). In particular, the MR study is an effective method for assessing causal relationships when it is unethical to conduct an RCT, for instance, when determining the causal association between two diseases (19). However, to our knowledge, no MR studies investigating the causal effect of GERD on anxiety disorders and depression have been reported. Hence, by conducting an MR study, we would be able to determine the causal impact of GERD on anxiety and depression, which would provide theoretical evidence for conducting psychological treatment for GERD patients, thus preventing the development of anxiety disorders and depression in the future.

## 2. Materials and methods

### 2.1. GWAS summary-level data of GERD, anxiety disorders, and depression

**FIGURE 1:**
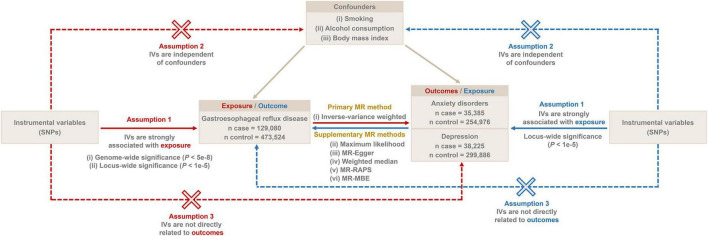
The overall flow chart of bidirectional MR study. SNP, single-nucleotide polymorphism; MR-RAPS, robust adjusted profile score; MR-MBE, mode-based estimate.

The overall flow chart of the bidirectional MR study is shown in Figure 1. The genome-wide association studies (GWAS) summary statistics for GERD were obtained from a recent genome-wide association meta-analysis study (12) that included 129,080 European GERD patients and 473,524 healthy controls (20). These data are available in the IEU Open GWAS Project database (21).^1^ Additionally, GWAS summary statistics for anxiety disorders and depression were extracted from the FinnGen consortium R8 release (22).^2^ The data for anxiety disorders were obtained from 290,361 individuals (35,385 cases and 254,976 controls), while the data for depression came from 338,111 individuals (38,225 cases and 299,886 controls). Table 1 provides details of the GWAS summary-level data of exposure and outcome analyzed in this MR study. All data analyzed in this study were obtained from publicly available databases in which ethical approval was obtained for each cohort, and informed consent was obtained from all participants prior to participation.

**TABLE 1:** Details of the GWAS summary-level data.

Traits	*N* case	*N* control	Population	Data accession address
GERD	129,080	473,524	European	https://gwas.mrcieu.ac.uk/
Anxiety disorders	35,385	254,976	European	https://r8.finngen.fi/
Depression	38,225	299,886	European	https://r8.finngen.fi/

GERD, gastroesophageal reflux disease.

### 2.2. Selection of instrumental variables

We selected IVs based on three generally recognized assumptions: (i) IVs need to be strongly associated with exposure, (ii) IVs are independent of confounders, and (iii) IVs are solely related to outcomes through exposure without a direct association with outcomes (17). We first screened the IVs for MR analysis using a genome-wide significance threshold (*P* < 5e−8). In addition, to increase the confidence of our findings, we additionally screened more IVs under a locus-wide significance threshold (*P* < 1e−5) for a secondary MR analysis. The SNPs within a window size of 10,000 kb were pruned under the threshold of *r*^2^ < 0.001 to mitigate linkage disequilibrium (LD), thus ensuring the independence of each IV. Smoking, alcohol consumption, and body mass index (BMI) might be potential confounders influencing GERD, anxiety disorders, and depression (23–27). Therefore, we retrieved SNPs associated with these confounders (*P* < 5e−8) from the IEU Open GWAS project database and excluded them from the IVs. The accession number of these confounders is shown in Table 2. Then, palindromic SNPs, outcome-related SNPs (*P* < 0.05), and SNPs not present in outcome GWAS summary data were removed from the IVs. Finally, we calculated the *F*-statistic of IVs to assess the degree of weak instrumental bias. Only IVs with *F* > 10 were retained to avoid bias caused by weak IVs (28).

**TABLE 2:** Source of the confounders.

Confounders	Source
Smoking	https://gwas.mrcieu.ac.uk/datasets/ieu-b-4877/
	https://gwas.mrcieu.ac.uk/datasets/ukb-b-223/
Alcohol consumption	https://gwas.mrcieu.ac.uk/datasets/ieu-b-73/
	https://gwas.mrcieu.ac.uk/datasets/ukb-b-5779/
BMI	https://gwas.mrcieu.ac.uk/datasets/ieu-b-40/
	https://gwas.mrcieu.ac.uk/datasets/ukb-b-19953/

BMI, body mass index.

### 2.3. Statistical methods

The inverse-variance weighted (IVW) method was used as the primary analytical method for estimating causal effects, which is an extension of the Wald ratio estimator based on the principles of meta-analysis (29). The significance threshold was set at *P* < 0.05, and the results of causal associations were presented as odds ratios (OR) and 95% confidence intervals (95% CI). To further evidence the stability and directionality of the results, in addition to the IVW method, five additional MR methods [maximum likelihood, MR-Egger, weighted median, robust adjusted profile score (MR-RAPS), and mode-based estimate (MR-MBE)] were performed to assess causal associations. The maximum likelihood is a traditional means which estimates probability distribution parameters by maximizing the likelihood function with low standard errors (30). The criterion for using the weighted median method is that at least 50% of the SNPs must satisfy the premise that they are valid IVs (31). The MR-Egger method provides unbiased estimates even in the presence of horizontal pleiotropy (32). MR-RAPS provides robust estimates in the presence of systematic and idiosyncratic pleiotropy (33). MR-MBE provides robust causal estimates when horizontal pleiotropy is present in most IVs and has a stronger effect on detecting causality than MR-Egger (34).

We then performed a series of sensitivity analyses. First, the MR Steiger test was performed to ensure that causal inferences were not biased by reverse causality (35). Next, Cochran’s Q test was used to assess heterogeneity. Then, the MR-Egger intercept and MR-PRESSO global tests were used to detect horizontal pleiotropy (36, 37). Finally, the leave-one-out sensitivity analysis was performed to evaluate the robustness of the results.

For the above analysis process, we first performed the MR analysis based on the IVs screened under the genome-wide significance threshold (*P* < 5e−8). Subsequently, we performed a secondary MR analysis based on more IVs screened under the locus-wide significance threshold *(P* < 1e−5) to validate the findings. All analyses in this study were performed based on R software (version 4.2.1). The “TwoSampleMR” R package (38),^3^ the “MendelianRandomization” R package (39),^4^ and the “’MRPRESSO” R package^5^ (37) were used in our MR study.

### 2.4. Reverse Mendelian randomization analysis

We further performed a reverse MR analysis to assess whether anxiety disorders or depression causally affect GERD. Since the number of SNPs satisfying the genome-wide significance threshold (*P* < 5e−8) in the GWAS summary-level dataset for anxiety and depression was extremely limited, we screened SNPs satisfying the locus-wide significance threshold (*P* < 1e−5) threshold as IVs associated with anxiety or depression. Subsequently, (i) screening of IVs, (ii) MR analysis, and (iii) sensitivity analyses were performed as described in sections “2.2. Selection of instrumental variables” and “2.3. Statistical methods.”

### 2.5. Multivariable Mendelian randomization analysis

Finally, a multivariable MR (MVMR) analysis was implemented for significant exposure-outcome pairs identified by univariate MR analysis. Specifically, three confounders, smoking (IEU GWAS ID: “ieu-b-4877”), alcohol consumption (IEU GWAS ID: “ukb-b-5779”), and BMI (IEU GWAS ID: “ukb-b-19953”), were included for MVMR analysis. After combining the GWAS summary level datasets of exposure and the three confounders, it should be ensured that each IV is strongly correlated (*P* < 5e−8) with at least one or more of the exposure or the three confounders. Then, the SNPs within a window size of 10,000 kb were pruned under the threshold of *r*^2^ < 0.001 to mitigate LD. Finally, after excluding palindromic SNPs, outcome-related SNPs (*P* < 0.05), and SNPs not present in outcome GWAS summary data, we used the IVW method to assess causal effects after adjusting for confounders.

## 3. Results

### 3.1. Results of MR analysis using IVs screened based on the genome-wide significance

The MR results of this section were based on the IVs screened under the genome-wide significance threshold (*P* < 5e−8).

First, a total of 41 SNPs associated with confounders (smoking, alcohol consumption, and BMI) were excluded. Subsequently, after excluding SNPs not present in the outcome, outcome-related SNPs, and palindromic SNPs, we assessed the causal effects of GERD on anxiety disorders and depression based on 15 and 16 IVs, respectively. Details of the confounders/outcomes-related SNPs are available in Supplementary Table 1, and details of the IVs for MR analysis are presented in Supplementary Table 2. In addition, the *F*-statistics of all IVs ranged from 29.75 to 50.68.

**FIGURE 2:**
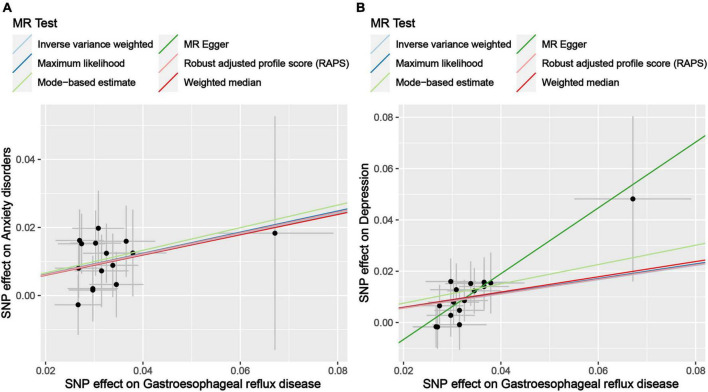
Scatter plot of genetic correlations of exposure and outcome based on the IVs screened under the genome-wide significance threshold. **(A)** Scatter plot of genetic correlations of GERD and anxiety disorders. **(B)** Scatter plot of genetic correlations of GERD and depression.

The results of the MR analysis based on the IVs screened under the genome-wide significance threshold are presented in Table 3. The MR results suggest a causal effect of GERD on anxiety disorders and depression. Specifically, the MR results of IVW indicated that GERD significantly increased the risk of anxiety disorders (OR = 1.35, 95% CI 1.15–1.59, *P* = 2.25 × 10^–4^) and depression (OR = 1.32, 95% CI: 1.15–1.52, *P* = 1.26 × 10^–4^) (Table 3). In addition, similar causal estimation results were derived from five other MR methods, including maximum likelihood, MR-Egger, weighted median, MR-RAPS, and MR-MBE (Table 3 and Figure 2).

**TABLE 3:** Mendelian randomization results of the causal effect of GERD on anxiety disorders and depression (based on the IVs screened under the genome-wide significance threshold).

Exposure	Outcome	*n* SNP	Method	OR (95% CI)	*P*-value
GERD	Anxiety disorders	15	IVW	1.35 (1.15, 1.59)	2.25E−04
			Maximum likelihood	1.36 (1.16, 1.61)	2.13E−04
			MR-Egger	1.35 (0.42, 4.36)	6.21E−01
			Weighted median	1.35 (1.10, 1.65)	4.59E−03
			MR-RAPS	1.36 (1.15, 1.61)	3.99E−04
			MR-MBE	1.39 (0.98, 1.98)	6.18E−02
GERD	Depression	16	IVW	1.32 (1.15, 1.52)	1.26E−04
			Maximum likelihood	1.33 (1.15, 1.54)	1.13E−04
			MR-Egger	3.61 (1.22, 10.63)	3.57E−02
			Weighted median	1.35 (1.11, 1.63)	2.01E−03
			MR-RAPS	1.32 (1.14, 1.54)	2.30E−04
			MR-MBE	1.46 (1.08, 1.96)	1.29E−02

SNP, single-nucleotide polymorphism; OR, odds ratio; CI, confidence interval; GERD, gastroesophageal reflux disease; IVW, inverse variance weighted; MR-RAPS, robust adjusted profile score; MR-MBE, mode-based estimate.

Subsequently, sensitivity analyses were performed to assess the robustness of the MR results. First, the MR Steiger test indicated that the inferred causal direction between exposure (GERD) and outcomes (anxiety disorder and depression) was “TRUE.” Then, Cochran’s Q test suggested that there was no heterogeneity among the IVs in our MR analysis (*P* > 0.05) (Table 4). In addition, the results of both the MR-Egger intercept test and the MR-PRESSO global test suggested that the MR analysis did not suffer from any potential influence of horizontal pleiotropy (*P* > 0.05) (Table 5). Finally, the leave-one-out sensitivity analysis confirmed the robustness of the MR results since there were no leading SNPs that could drastically affect the results after being eliminated (Supplementary Figure 1).

**TABLE 4:** Results of heterogeneity by the Cochran’s Q test (based on the IVs screened under the genome-wide significance threshold).

Exposure	Outcome	Method	Cochran’s Q test		
			**Q**	**Q_df**	**Q_pval**
GERD	Anxiety disorders	Inverse variance weighted	6.345	14	0.957
		MR-Egger	6.345	13	0.933
GERD	Depression	Inverse variance weighted	6.988	15	0.958
		MR-Egger	3.613	14	0.997

GERD, gastroesophageal reflux disease.

**TABLE 5:** Results of horizontal pleiotropy by the MR-Egger intercept test and MR-PRESSO global test (based on the IVs screened under the genome-wide significance threshold).

Exposure	Outcome	MR-Egger intercept test			MR-PRESSO global test	
		**Intercept**	**SE**	***P*-value**	**RSS obs**	***P*-value**
GERD	Anxiety disorders	1.64E−05	0.019	0.999	7.271	0.960
GERD	Depression	−0.032	0.018	0.088	7.839	0.964

GERD, gastroesophageal reflux disease; SE, standard error; RSS, residual sum of squares.

### 3.2. Results of MR analysis using IVs screened based on the locus-wide significance

In order to increase the confidence of the MR results, a secondary MR analysis was performed using IVs screened based on the locus-wide significance threshold (*P* < 1e−5), for which the following section shows the results.

First, a total of 92 SNPs associated with confounders (smoking, alcohol consumption, and BMI) were excluded. Subsequently, after excluding SNPs not present in the outcome, outcome-related SNPs, and palindromic SNPs, we assessed the causal effects of GERD on anxiety disorders and depression based on 75 and 79 IVs, respectively. Details of the confounders/outcomes-related SNPs are available in Supplementary Table 3, and details of the IVs for MR analysis are presented in Supplementary Table 4. In addition, the *F*-statistics of all IVs ranged from 19.67 to 50.68.

**FIGURE 3:**
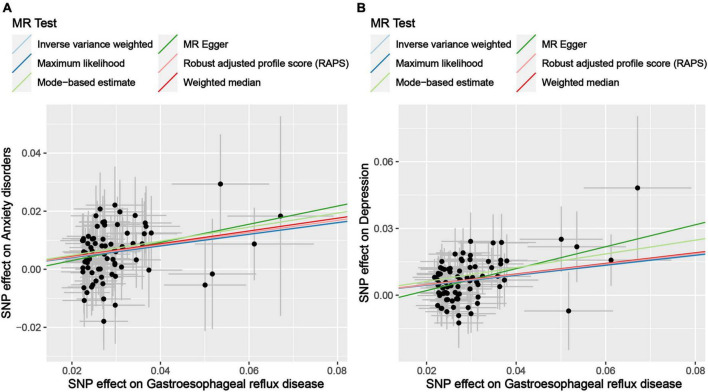
Scatter plot of genetic correlations of exposure and outcome based on the IVs screened under the locus-wide significance threshold. **(A)** Scatter plot of genetic correlations of GERD and anxiety disorders. **(B)** Scatter plot of genetic correlations of GERD and depression.

The results of the MR analysis based on the IVs screened under the locus-wide significance threshold are presented in Table 6. Interestingly, similar to the MR results in the previous section, the MR results in this section remained indicative of a causal effect of GERD on anxiety and depression. Specifically, the MR results of IVW indicated that GERD significantly increased the risk of anxiety disorders (OR = 1.22, 95% CI: 1.13–1.32, *P* = 1.19 × 10^–6^) and depression (OR = 1.25, 95% CI: 1.16–1.34, *P* = 1.81 × 10^–9^) (Table 6). In addition, similar causal estimation results were derived from five other MR methods, including maximum likelihood, MR-Egger, weighted median, MR-RAPS, and MR-MBE (Table 6 and Figure 3).

**TABLE 6:** Results of the causal effect of GERD on anxiety disorders and depression (based on the IVs screened under the locus-wide significance threshold).

Exposure	Outcome	*n* SNP	Method	OR (95% CI)	*P*-value
GERD	Anxiety disorders	75	IVW	1.22 (1.13, 1.32)	1.19E−06
			Maximum likelihood	1.22 (1.13, 1.33)	1.50E−06
			MR-Egger	1.37 (0.93, 2.01)	1.16E−01
			Weighted median	1.25 (1.11, 1.39)	1.17E−04
			MR-RAPS	1.23 (1.13, 1.34)	1.27E−06
			MR-MBE	1.27 (0.99, 1.63)	5.65E−02
GERD	Depression	79	IVW	1.25 (1.16, 1.34)	1.81E−09
			Maximum likelihood	1.25 (1.16, 1.35)	2.59E−09
			MR-Egger	1.64 (1.16, 2.32)	6.75E−03
			Weighted median	1.27 (1.14, 1.41)	7.04E−06
			MR-RAPS	1.26 (1.17, 1.36)	2.89E−09
			MR-MBE	1.36 (1.08, 1.72)	8.66E−03

SNP, single-nucleotide polymorphism; OR, odds ratio; CI, confidence interval; GERD, gastroesophageal reflux disease; IVW, inverse variance weighted; MR-RAPS, robust adjusted profile score; MR-MBE, mode-based estimate.

Subsequently, sensitivity analyses were performed to assess the robustness of the MR results. First, the MR Steiger test indicated that the inferred causal direction between exposure (GERD) and outcome (anxiety disorder and depression) was “TRUE.” Then, Cochran’s Q test suggested that there was no heterogeneity among the IVs in our MR analysis (*P* > 0.05) (Table 7). In addition, the results of both the MR-Egger intercept test and the MR-PRESSO global test suggested that the MR analysis did not suffer from any potential influence of horizontal pleiotropy (*P* > 0.05) (Table 8). Finally, the leave-one-out sensitivity analysis confirmed the robustness of the MR results since there were no leading SNPs that could drastically affect the results after being eliminated (Supplementary Figure 2).

**TABLE 7:** Results of heterogeneity by the Cochran’s Q test (based on the IVs screened under the locus-wide significance threshold).

Exposure	Outcome	Method	Cochran’s Q test		
			**Q**	**Q_df**	**Q_pval**
GERD	Anxiety disorders	Inverse variance weighted	47.140	74	0.994
		MR-Egger	46.788	73	0.993
GERD	Depression	Inverse variance weighted	52.773	78	0.987
		MR-Egger	50.320	77	0.992

GERD, gastroesophageal reflux disease.

**TABLE 8:** Results of horizontal pleiotropy by the MR-Egger intercept test and MR-PRESSO global test (based on the IVs screened under the locus-wide significance threshold).

Exposure	Outcome	MR-Egger intercept test			MR-PRESSO global test	
		**Intercept**	**SE**	***P*-value**	**RSS obs**	***P*-value**
GERD	Anxiety disorders	−0.003	0.006	0.555	48.350	0.996
GERD	Depression	−0.008	0.005	0.121	54.069	0.989

GERD, gastroesophageal reflux disease; SE, standard error; RSS, residual sum of squares.

### 3.3. Results of reverse Mendelian randomization analysis

We performed a reverse MR analysis to assess whether anxiety disorders or depression causally affect GERD. First, 12 and 9 SNPs associated with confounders (smoking, alcohol consumption, and BMI) were excluded from the IVs of anxiety disorders and depression, respectively. Subsequently, after excluding SNPs not present in the outcome, outcome-related SNPs, and palindromic SNPs, we assessed the causal effects of anxiety disorders and depression on GERD based on 16 and 16 IVs, respectively. Details of the confounders/outcomes-related SNPs are available in Supplementary Table 5, and details of the IVs for reverse MR analysis are presented in Supplementary Table 6. In addition, the *F*-statistics of all IVs ranged from 19.71 to 35.67.

All MR methods showed no causal relationship between anxiety or depression and GERD risk (*P* > 0.05) (Table 9). Cochran’s Q test showed that the reverse MR analysis was not influenced by heterogeneity (*P* > 0.05) (Table 10). In addition, the MR-Egger intercept test and MR-PRESSO global test indicated that the reverse MR analysis was not influenced by water product pleiotropy (*P* > 0.05) (Table 11). Finally, the leave-one-out sensitivity analysis confirmed the robustness of the reverse MR results (Supplementary Figure 3).

**TABLE 9:** Reverse MR results of the causal effect of anxiety disorders and depression on GERD.

Exposure	Outcome	*n* SNP	Method	OR (95% CI)	*P*-value
Anxiety disorders	GERD	16	IVW	1.03 (0.98, 1.09)	0.24
			Maximum likelihood	1.04 (0.98, 1.10)	0.23
			MR-Egger	1.06 (0.88, 1.28)	0.57
			Weighted median	1.03 (0.96, 1.11)	0.43
			MR-RAPS	1.04 (0.98, 1.10)	0.22
			MR-MBE	1.02 (0.90, 1.17)	0.75
Depression	GERD	16	IVW	1.05 (0.99, 1.12)	0.08
			Maximum likelihood	1.06 (0.99, 1.12)	0.08
			MR-Egger	0.87 (0.66, 1.16)	0.37
			Weighted median	1.08 (0.99, 1.17)	0.08
			MR-RAPS	1.06 (1.00, 1.13)	0.06
			MR-MBE	1.09 (0.95, 1.25)	0.22

SNP, single-nucleotide polymorphism; OR, odds ratio; CI, confidence interval; GERD, gastroesophageal reflux disease; IVW, inverse variance weighted; MR-RAPS, robust adjusted profile score; MR-MBE, mode-based estimate.

**TABLE 10:** Results of heterogeneity by the Cochran’s Q test in reverse MR analysis.

Exposure	Outcome	Method	Cochran’s Q test		
			**Q**	**Q_df**	**Q_pval**
Anxiety disorders	GERD	Inverse variance weighted	10.675	15	0.775
		MR-Egger	10.612	14	0.716
Depression	GERD	Inverse variance weighted	13.352	15	0.575
		MR-Egger	11.582	14	0.640

GERD, gastroesophageal reflux disease.

**TABLE 11:** Results of horizontal pleiotropy by the MR-Egger intercept test and MR-PRESSO global test in reverse MR analysis.

Exposure	Outcome	MR-Egger intercept test			MR-PRESSO global test	
		**Intercept**	**SE**	***P*-value**	**RSS obs**	***P*-value**
Anxiety disorders	GERD	−0.001	0.005	0.806	12.051	0.781
Depression	GERD	0.009	0.007	0.205	15.062	0.595

GERD, gastroesophageal reflux disease; SE, standard error; RSS, residual sum of squares.

### 3.4. Results of multivariable Mendelian randomization analysis

**FIGURE 4:**
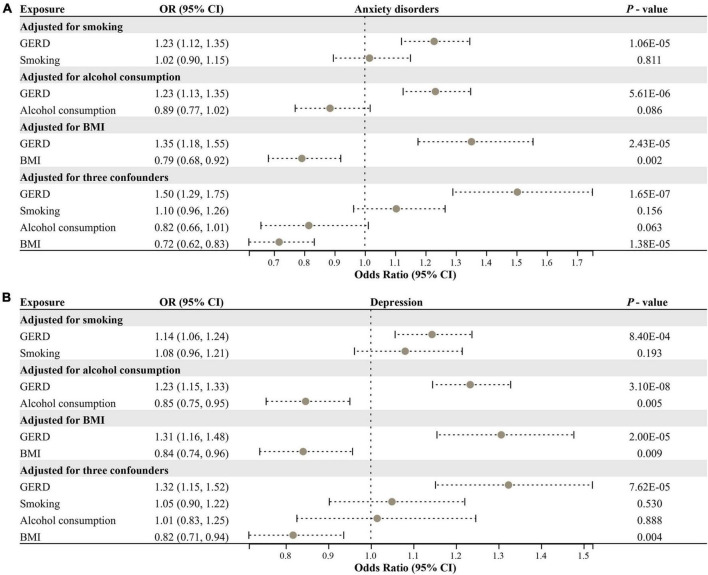
Multivariable MR analysis for assessing the causal effect of GERD on anxiety disorders and depression. **(A)** Forest plot of the causal effect of GERD on anxiety disorders after adjusting for confounders. **(B)** Forest plot of the causal effect of GERD on depression after adjusting for confounders. OR, odds ratio; CI, confidence interval; GERD, gastroesophageal reflux disease; BMI, body mass index.

We performed an MVMR analysis to assess the causal effect of GERD on anxiety and depression after adjusting for three confounding factors (smoking, alcohol consumption, and BMI). MVMR analysis identified that: for anxiety disorders, after adjusting for smoking (OR = 1.23, 95% CI: 1.12–1.35, *P* = 1.06E−05), alcohol consumption (OR = 1.23, 95% CI: 1.13–1.35, *P* = 5.61E−06), BMI (OR = 1.35, 95% CI: 1.18–1.55, *P* = 2.43E−05), and all of these three confounders (OR = 1.50, 95% CI: 1.29–1.75, *P* = 1.65E−07), GERD remained causally related to anxiety disorders risk and had a more substantial effect than the causal relationship identified by univariate MR (Figure 4A). For depression, after adjusting for smoking (OR = 1.14, 95% CI: 1.06–1.24, *P* = 8.40E−04), alcohol consumption (OR = 1.23, 95% CI: 1.15–1.33, *P* = 3.10E−08), BMI (OR = 1.31, 95% CI: 1.16–1.48, *P* = 2.00E−05), and all of these three confounders (OR = 1.32, 95% CI: 1.15–1.52, *P* = 7.62E−05), GERD remained causally associated with depression risk, with effects remaining consistent with univariate MR results (Figure 4B). Overall, GERD is causally associated with an increased risk of anxiety disorders and depression.

## 4. Discussion

In this study, we performed a bidirectional MR analysis using multiple MR approaches and ultimately demonstrated that genetically predicted GERD can significantly elevate the risk of anxiety disorders and depression. In addition, these associations were robust in sensitivity analysis with no heterogeneity and pleiotropy detected. Furthermore, the above results were consistent both in MR analyses using IVs screened based on the genome-wide significance thresholds and using IVs screened based on the locus-wide significance thresholds, increasing the confidence of our results. Nevertheless, the reverse MR analysis identified that depression or anxiety did not appear to have a causal effect on GERD. To our knowledge, this is the first study to explore the causal association between GERD and anxiety disorders/depression by conducting an MR analysis with large-scale GWAS summary-level data, which enriched and refined the results of previous related findings.

Previous observational studies have implied a probable relationship between GERD and anxiety disorders/depression. Choi et al. found that GERD patients had higher levels of anxiety and depression compared to healthy controls, especially in the subgroup of patients with non-erosive reflux disease (8). Likewise, a prospective cohort study involving Australian men identified elevated anxiety and depression levels in GERD patients (40). A cohort study conducted by You et al. through the Taiwan National Health Insurance Research Database revealed a significantly higher prevalence of anxiety and depression in the GERD cohort compared to the control cohort, as well as a higher rate of new morbidity in the follow-up durations (10). In addition, the severity of GERD also contributes to the incidence of psychiatric disorders. For instance, Wu et al. reported that depressive symptoms in GERD patients could be improved after PPI therapy (41). Similarly, Kimura et al. evaluated anxiety and depression scores in GERD patients treated with PPIs and found significantly higher scores in the PPI partial response group compared with the PPI response group (9). Furthermore, Quach et al. found that the long duration of reflux symptoms was a risk factor for the development of depression in GERD patients (42). While these observational studies cannot explain causal effects, they provide sufficient evidence for an association between GERD and anxiety disorders/depression. Using the MR study, we demonstrated that GERD might increase the incidence of anxiety disorders and depression, which strengthens the findings of these prior observational investigations.

The MR study is an innovative approach to deducing causality. Compared to conventional observational research, MR studies eliminate confounding variables and reverse causality. Compared to RCTs, MR studies are more efficient and have no ethical restrictions on their implementation. The selection of IVs in our MR study was strictly based on the three main assumptions of the MR study. For assumption 1, we adopted a genome-wide significance threshold (*P* < 5e−8) or locus-wide significance threshold (*P* < 1e−5) to screen SNPs associated with GERD as IVs. In addition, we eliminated the LD of IVs. Furthermore, the *F*-statistic of IVs is greater than 10. For assumption 2, we excluded SNPs strongly associated with confounders (*P* < 5 × 10^–8^) from the IVs. Finally, for assumption 3, SNPs associated with outcomes were removed from the IVs. IVW, the most predominant analytical method in MR studies, suggested that GERD considerably raised the risk of anxiety disorders (OR = 1.35, 95% CI: 1.15–1.59, *P* = 2.25 × 10^–4^) and depression (OR = 1.32, 95% CI: 1.15–1.52, *P* = 1.26 × 10^–4^) using IVs screened based on the genome-wide significance threshold. In addition, the inference findings from five other MR approaches were congruent with the IVW’s result. Although the MR-Egger finding was not statistically significant (*P* > 0.05) due to the method’s limited power and high Type 1 error rates (32), the estimated effect remained in the same direction (OR > 1). Subsequently, we conducted various sensitivity tests that further demonstrated the validity of the findings. Interestingly, the above results were consistent in the secondary MR analysis using IVs screened based on the locus-wide significance threshold.

Several presumptions may explain the increased risk of anxiety disorders and depression caused by GERD. First, increasing global inflammation levels may contribute to the increased risk of anxiety disorders and depression induced by GERD (10). Studies have reported that the esophageal mucosa of GERD patients generates more cytokines and chemokines, including interleukin-6, interleukin-8, interleukin-1 beta, tumor necrosis factor-alpha, platelet-activating factor, and reactive oxygen species, consequently elevating the inflammation levels of the central nervous system (10). In addition, chronic and moderate inflammation in the peripheral circulation and brain have been shown to contribute to the development of anxiety disorders and depression (43, 44). Second, sleep disorders may mediate GERD-induced anxiety disorder/depression. Patients with GERD frequently suffer from more severe sleep disorders since nighttime reflux episodes are commonly accompanied by conscious arousals, which could provoke sleep disorders (11, 12). In addition, frequent awakenings activate the neuroendocrine system, including the autonomic nervous system (ANS) and the hypothalamic-pituitary-adrenal axis, thereby increasing sympathetic activation and thus exacerbating sleep disorders (11). In addition, acid reflux stimulates the vagal nerve and triggers frequent bronchial constriction, leading to the narrowing of the diameter of the airway and aggravating sleep disorder (45). In a prospective study, chronic insomnia was identified as a risk factor for anxiety and depression (46). Furthermore, sleep deprivation induces nociceptive hypersensitivity of the esophageal mucosa in response to acid (47). Therefore, it is necessary to maintain nighttime gastric protection therapy for GERD patients, thus improving sleep quality to reduce the risk of subsequent anxiety disorders and depression.

The present study has some strengths: First, to the best of our knowledge, this is the first MR investigation to evaluate the causal relationship between GERD and anxiety disorders/depression. Second, the present MR analysis was conducted using separate summary-level data from large-scale GWAS, which boosts the confidence of inference due to the substantial sample size. Third, numerous MR methods and sensitivity analyses were employed to enhance the credibility of the findings.

Nevertheless, the present study has some limitations. First, the original GWAS summary-level data analyzed in this study were derived from European populations; hence, the findings may not be applicable to other ethnicities. Second, a stratified analysis based on general factors such as age and gender was unavailable due to the limitations of GWAS summary data. Third, it is difficult to ensure that the results are entirely independent of the horizontal polymorphism effect. Therefore, a series of sensitivity analyses were conducted to demonstrate the reliability of the results.

## 5. Conclusion

We have provided evidence that genetically predicted GERD increases the risk of anxiety disorders and depression. Therefore, symptomatic treatment for GERD patients should be accompanied by adequate psychological support to avoid the development of anxiety disorders and depression.

## Data availability statement

The original contributions presented in this study are included in the article/Supplementary material, further inquiries can be directed to the corresponding author.

## Ethics statement

All data analyzed in this study were obtained from publicly available databases in which ethical approval was obtained for each cohort, and informed consent was obtained from all participants prior to participation. The patients/participants provided their written informed consent to participate in this study.

## Author contributions

YZ designed the study, analyzed the data, and wrote the manuscript. SC assisted in analyzing the data and revising the manuscript. HY critically read and edited the manuscript. All authors contributed to the article and approved the submitted version.

## Footnotes

1 https://gwas.mrcieu.ac.uk/ accession number: “ebi-a-GCST90000514”.

2
https://r8.finngen.fi/

3
https://mrcieu.github.io/TwoSampleMR/

4
https://cran.r-project.org/web/packages/MendelianRandomization/index.html

5
https://github.com/rondolab/MR-PRESSO
