# Supplementary material for: The causal role of gastroesophageal reflux disease in anxiety disorders and depression: A bidirectional Mendelian randomization study
Source: Front Psychiatry. 2023 Feb 22;14:1135923. doi: 10.3389/fpsyt.2023.1135923 (PMC9992201; doi:10.3389/fpsyt.2023.1135923)
Supplement: Supplementary file 1 [file Data_Sheet_1.PDF]

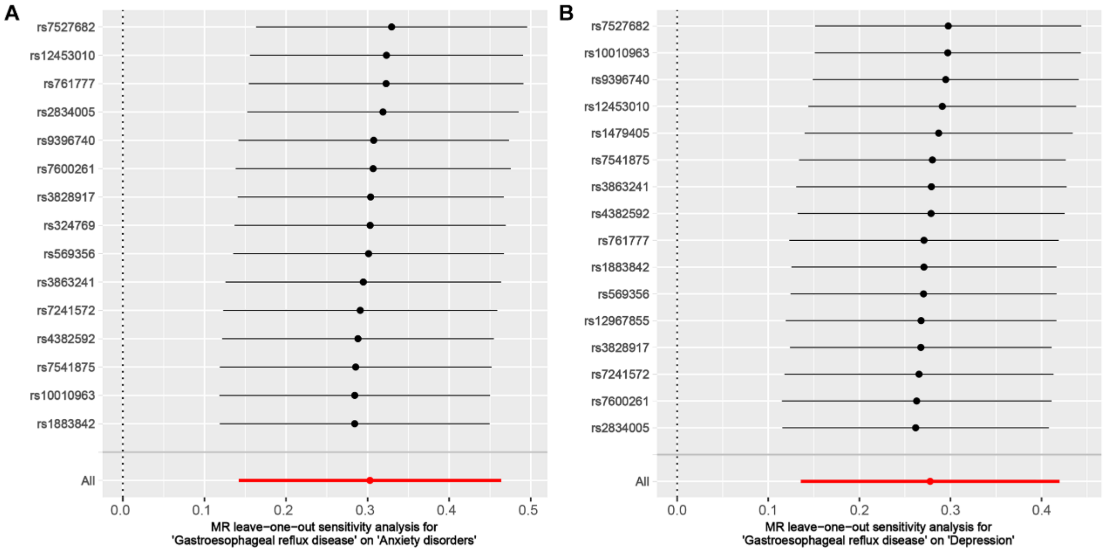

Supplementary Figure 1. The leave-one-out sensitivity analysis based on the IVs screened under the genome-wide significance threshold. (A) leave-one-out sensitivity analysis for gastroesophageal reflux disease on anxiety disorders. (B) leave-one-out sensitivity analysis for gastroesophageal reflux disease on depression.

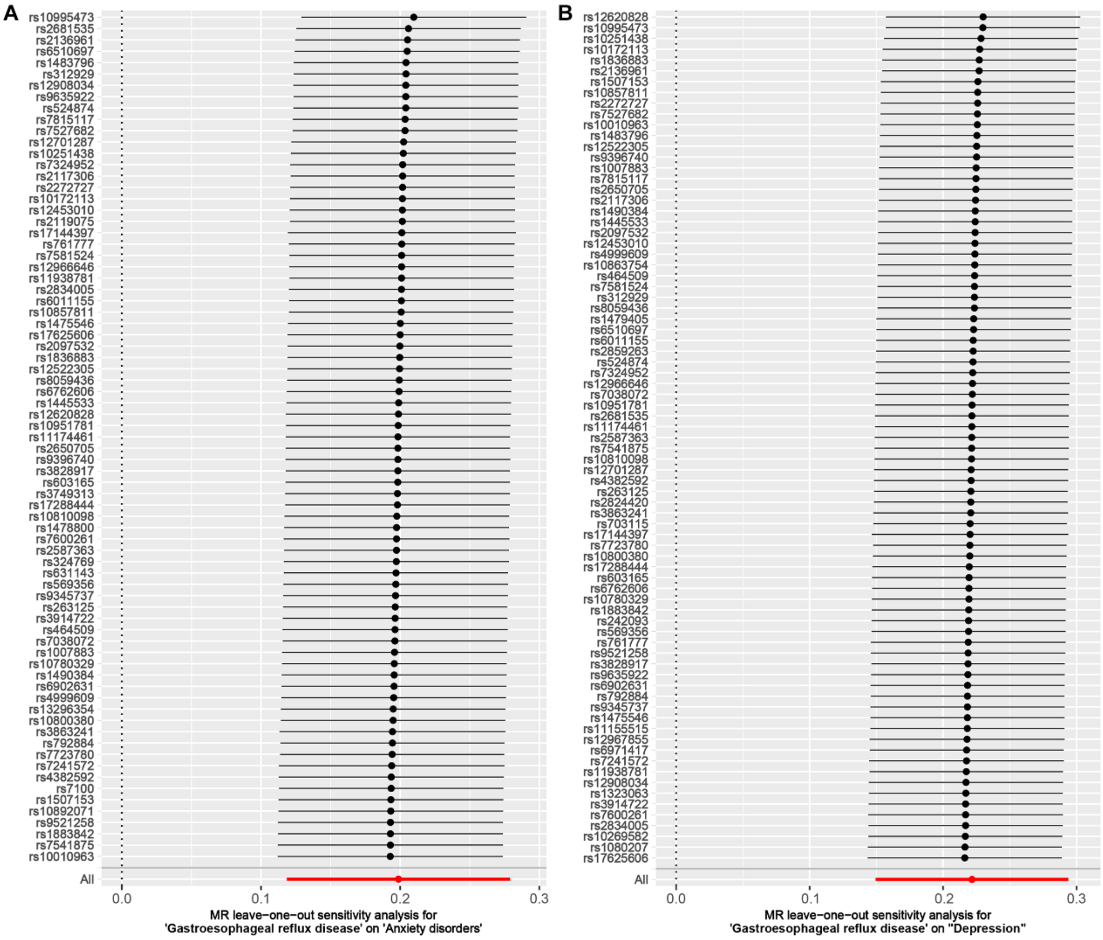

Supplementary Figure 2. The leave-one-out sensitivity analysis based on the IVs screened under the locus-wide significance threshold. (A) leave-one-out sensitivity analysis for gastroesophageal reflux disease on anxiety disorders. (B) leave-one-out sensitivity analysis for gastroesophageal reflux disease on depression.

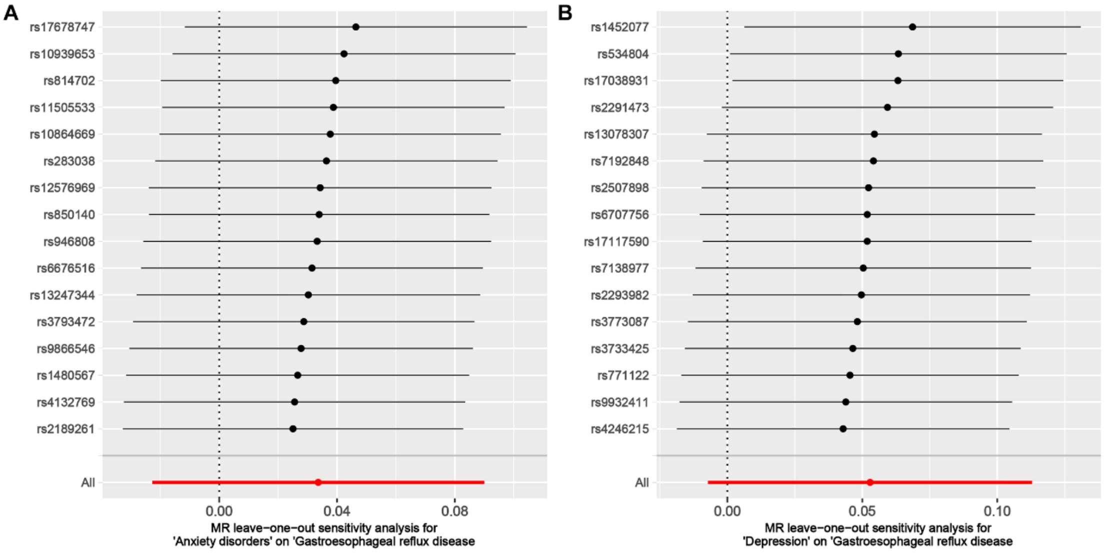

Supplementary Figure 3. The leave-one-out sensitivity analysis in reverse MR analysis. (A) leave-one-out sensitivity analysis for anxiety disorders on gastroesophageal reflux disease. (B) leave-one-out sensitivity analysis for depression on gastroesophageal reflux disease.
